# F232. A PHASE 3 STUDY TO DETERMINE THE ANTIPSYCHOTIC EFFICACY AND SAFETY OF ALKS 3831 IN ADULT PATIENTS WITH ACUTE EXACERBATION OF SCHIZOPHRENIA

**DOI:** 10.1093/schbul/sby017.763

**Published:** 2018-04-01

**Authors:** David McDonnell, Steven G Potkin, Adam Simmons, Ying Jiang, Lauren DiPetrillo, Bernard Silverman

**Affiliations:** 1 Alkermes, Inc; 2 University of California, Irvine

## Abstract

**Background:**

ALKS 3831, currently under development for the treatment of schizophrenia, is composed of a flexible dose of olanzapine (OLZ) and a fixed dose of 10 mg of samidorphan. In a Phase 2 study, ALKS 3831 mitigated OLZ-associated weight gain and exhibited antipsychotic efficacy similar to OLZ alone. This Phase 3 study assessed antipsychotic efficacy and safety of ALKS 3831 in patients with acute exacerbation of schizophrenia.

**Methods:**

This was an international (USA, Ukraine, Serbia, and Bulgaria), 4-week, randomised, double-blind, active and placebo (PBO)-controlled study of ALKS 3831 in patients with acute exacerbation of schizophrenia (ClinicalTrials.gov: NCT02634346). Eligible patients (N=403) were randomised 1:1:1 to receive either ALKS 3831, OLZ, or PBO. Patients were treated in an inpatient setting for the first 2 weeks of the study and could be treated as inpatients or outpatients for the remaining 2 weeks. Patients were excluded if they received OLZ within 6 months prior to screening. Antipsychotic efficacy was assessed using the Positive and Negative Syndrome Scale (PANSS), Clinical Global Impression–Severity (CGI-S), and CGI–Improvement (CGI-I) scales. Safety and tolerability were assessed as adverse events (AEs).

**Results:**

Of 401 patients randomised and dosed to ALKS 3831, OLZ, and PBO, 91%, 89%, and 83% of patients, respectively, completed treatment. The most common reason for discontinuation was withdrawal by patient (6% in both the ALKS 3831 and PBO groups, and 7% in the OLZ group). Baseline characteristics were generally similar between groups; however, baseline mean body-mass index was higher in the OLZ group than in the ALKS 3831 group. Baseline mean ± standard deviation scores were 101.7 ± 11.9 for PANSS total score and 5.1 ± 0.7 for CGI-S score. The mean OLZ dose was 18.4 mg/day in both active treatment arms. Least squares (LS) mean difference ± standard error (SE) versus PBO from baseline to Week 4 in PANSS total score was –6.4 ± 1.8 (P<.001) for the ALKS 3831 group and –5.3 ± 1.8 (P=.004) for the OLZ group. LS mean difference ± SE vs PBO from baseline to Week 4 in CGI-S score was −0.38 ± 0.12 (P=.002) for the ALKS 3831 group and −0.44 ± 0.12 (P<.001) for the OLZ group. The percentage of patients with an improvement in PANSS response (≥30% improvement from baseline) at Week 4 was 60%, 54%, and 38% in the ALKS 3831, OLZ, and PBO groups, respectively. The percentage of patients with an improvement in CGI-I response (score of ≤2) at Week 4 was 58%, 51%, and 33% in the ALKS 3831, OLZ, and PBO groups, respectively. Discontinuation due to AEs was low in all groups. Common AEs (≥5%) included weight gain, somnolence, dry mouth, anxiety, headache, and schizophrenia.

**Discussion:**

ALKS 3831 demonstrated greater antipsychotic efficacy than PBO, as measured by the PANSS and CGI-S scale, and was similar to the active control, OLZ. The safety profile of ALKS 3831 was similar to OLZ.

